# Neuropsychological assessment through Coma Recovery Scale-Revised and Coma/Near Coma Scale in a sample of pediatric patients with disorder of consciousness

**DOI:** 10.1007/s00415-022-11456-6

**Published:** 2022-11-05

**Authors:** Susanna Frigerio, Erika Molteni, Katia Colombo, Valentina Pastore, Claudia Fedeli, Susanna Galbiati, Sandra Strazzer

**Affiliations:** 1 Scientific Institute, IRCCS E. Medea, Neurophysiatric Department, Bosisio Parini, Lecco Italy; 2 School of Biomedical Engineering and Imaging Sciences, and Centre for Medical Engineering, King’s College, London, SE1 7EU UK

**Keywords:** Coma Recovery Scale Revised (CRS-R), Coma/Near Coma Scale (CNCS), Children, Pediatric disorder of consiousness, Emegence from coma in children

## Abstract

**Background:**

The Coma Recovery Scale-Revised (CRS-R) has become a standard tool in assessing Disorders of consciousness (DoC) in adults. However, its measurement validity in pediatrics has only been ascertained in healthy cases. Increasing use of CRS-R in children with DoC imposes appropriate comparison against previously validated tools. The aims of the study were to describe the emergence to a conscious state (eMCS) in pediatric acquired brain injury (ABI); to explore the agreement between the CRS-R and Coma Near Coma Scale (CNCS) and to discuss the advantage of administering the CRS-R in pediatric age.

**Materials and methods:**

In this observational prospective study, 40 patients were recruited. Inclusion criteria were age 5 to 18 years, Glasgow Coma Scale (GCS) score ≤ 8 at the insult, and unresponsive wakefulness syndrome (UWS) or minimally conscious state (MCS) at admission. Patients were assessed with CRS-R, and CNCS was used as standard.

**Results:**

The agreement between scales was moderate (*r* = − 0.71). The analysis of the CRS-R domain scores also confirmed that decreasing CNCS levels (from a coma to eMCS) corresponded to concurrent increas of CRS-R scores in all domains. Moreover, CRS-R better defined patients’ status in the emergency phase from MCS. Conversely, CRS-R had lower DoC scoring ability in the presence of severe motor impairment.

**Conclusion:**

We show that CRS-R can track changes in DoC in children as young as 5 years old, and we provide evidence that the agreement with CNCS scores is good.

**Supplementary Information:**

The online version contains supplementary material available at 10.1007/s00415-022-11456-6.

## Introduction

Acquired brain injury (ABI) in pediatric age is leading cause of disability and death worldwide, with an estimated incidence of ~ 240/100 000 individuals per year [1, 2]. The most severe injuries can result in Disorders of Consciouness (DoC), which assessment is the main challenge for centers delivering acute and post-acute rehabilitation, [3, 4]. Assessment is especially arduous in young children, whose neurobehavioral responses are naturally unreliable due to the early developmental stage, [5].

Definitions of DoC are well established in adults, and features of each state of consciousness have been highlighted in diagnostic guidelines issued by the European, [6] and American Academies of Neurology (AAN), [7]. Classification of DoC based on behavioral features has evolved over the past 50 years, and currently includes: coma, unresponsive wakefulness syndrome, minimally conscious states plus and minus, covert cognition, and locked-in-state [8–10].

Several behavioral measures have been developed to assess DoC in adults, among which the Coma Near Coma Scale (CNCS), [11] and the Level of Cognitive Functioning Assessment Scale (LOCFAS), [12, 13]. However, the Coma Recovery Scale-Revised (CRS-R), [14] has emerged as the gold standard in the neurobehavioral assessment of adults with DoC, after demonstration of excellent content validity, and acknowledgment by the Aspen Neurobehavioral Conference Workgroup, [15].

The last Guideline Report by AAN, [7] suggests that, in the absence of evidence specific to pediatrics, it is reasonable to apply the diagnostic recommendations for adult populations.

The absence of specific indications for children with DoC has significant consequences. Assessment often relies, at least partially, on clinicians’ own experience, rather than objective clinical evaluation, [16]. Misdiagnosis can occur, leading to under- or overestimation of patient’s functioning and imprecise prognosis, [16, 17] with major implications for treatment decisions, [18].

Some measures for DoC designed on adult patients have been used or tested in children. Clinical agreement was found between CNCS and LOCFAS in 92 patients with diagnosis of UWS or MCS at 3 and 6 months after injury, [19]. Also, Pham et al. [20] evaluated the conscious state in children and young adults after brain injury using CNCS. Overall, tools designed for adults lack powered validation in pediatrics, and standardisation of use is largely insufficient in clinical practice.

The pediatric adaptation of the CRS-R (CRS-P) has been tested on typically developing young children, [21]; however, it has not yet received formal evaluation in children with DoC. As CRS-R is considered the gold standard in adults, and by virtue of its intrinsic capability to discriminate between UWS and MCS [22], we evaluated the scale in a cohort of pediatric patients with DoC aged 5 to 18 years. The main implication of CRS-R validation in pediatrics is that the clinical assessment of DoC, including prognosis, will gain comparability across the entire lifespan, thus reconciling studies in children and adults.

We experimentally apply CRS-R scale to describe the level of consciousness in a sample of children and adolescents with a severe acquired brain lesion and a DoC at admission. We describe the cohort through a second index scale (i.e., standard), the CNCS, previously tested in children [19], and we explore the agreement between the two instruments, also in relation to the different etiologies.

## Materials and methods

### Participants

From a cohort of 294 consecutive children and adolescents with acquired brain injury (ABI), admitted between 8 April, 2015 and 31 December, 2020, we recruited 97 patients with a diagnosis of either unresponsive wakefulness syndrome (UWS) or minimally conscious state (MCS). Diagnostic compliance was assessed as per the indications available when the study started, [7, 22].

Inclusion criteria were: (1) age at the first assessment between 5 and 18 years; (2) documented evidence of severe ABI of traumatic, anoxic, vascular, or infective etiology (Glasgow Coma Scale (GCS) ≤ 8); (3) time between the ABI and the first assessment < 12 months; (4) medical records sufficiently detailed to determine injury severity and neurological findings; (5) no documented history of neurological or developmental disorders (i.e., autism, learning or attention disorders) or previously acquired brain lesions, and (6) no pre-existing acute or chronic severe illnesses (e.g., cerebral tumor). Children younger than 5 years were not considered in agreement with a preview study [21]. It is recommended to use the pediatric form of CRS-R to assess this last age group.

### Measures

For each patient, the following clinical and medical data were collected: gender, age at event, age at the time of assessment, GCS score at event, Glasgow Outcome Scale—Extended” GOS-E, [23] at admission, clinical diagnosis of UWS or MCS, days of unconsciousness, neurosurgery, presence of motor impairments, assisted respiratory function/need of tracheotomy, presence of paroxysmal sympathetic hyperactivity episodes, presence of feeding disorders, presence of epilepsy and previous rehabilitation. Outcome data 1 year after the event were also available for 37 patients.

An experienced neuropsychologist administered a neurobehavioral evaluation at admission, consisting in the full administration of the CNCS (Supplementary material 4) and CRS-R within a maximum time frame of 5 days. The order of CNCS and CRS-R administrations was randomised through a computerised routine. During hospitalisation, each subject received a variable number of additional pairs of CNCS and CRS-R evaluations (within 5 days), depending on their clinical status.

Based on previous work, [19] and to compare assessments effected through CNCS and CRS-R on a common ground, the theoretical framework linking the CNCS levels to the clinical status through the scale items was extended to the CRS-R domains (Supplementary Table 1). The clinical status was labelled as: (1) coma; (2) unresponsive wakefulness syndrome (UWS)/ vegetative state (VS); (3) minimally conscious state minus (MCS−); (4) minimally conscious state plus (MCS +); and (5) emergence to a conscious state (eMCS). The distinction between MCS− and MCS + was based on motor requests (command responsivity or command following) and not on verbal performance, because previous work, [19, 21] observed that the verbal responsivity is less reliable marker than the motor responsivity in young children. Preliminary full characterisation of CNCS and CRS-R instruments was conducted, based on Item Response Theory (IRT) and through Graded Response Models (GRM, Supplementary Material 5).

The study (NCT04499092, registered at https://clinicaltrials.gov) was approved by the local research Ethics Committee (Institutional Review Board) on Ricerca Corrente 2015–2018 and 2019–2022 (Italian Ministry of Health) and registered with the code Id. n.27/15-CE (8/4/15) and n. 607 Rev.1–v.1 (25.02.2019). All the caregivers gave their informed consent in line with the Declaration of Helsinki and subsequent amendments.

### Data analysis

#### Descriptive statistics and correlation between the CRS-R and the CNCS scales

Data were described through either parametric or non-parametric statistics, based on its distributions (Table 1). Statistical analysis was performed using SPSS Statistics 24 (SPSS.24, Inc, Chicago, IL) and Python [24] v3.8.5. Significance was assumed at a *p* value < 0.001 if not specified otherwise.

**Table 1:** Scores correlation between CRS-R and CNCS assessments, in corresponding sensory and motor domains

CRS-R domain	CNCS item(s)	Spearman’s correlation	*p* value
Auditory Consistent movement to command Reproducible movement to command Localization to sound Auditory startle None	Auditory Eye opening or orientation toward sound	− 0.71	< 0.00001
Visual Object Recognition Object Localization Visual Pursuit Fixation Visual Startle None	Visual Fixation or avoidance	− 0.79	< 0.00001
	Visual Fixation and tracking		
Motor Functional Object Use Automatic motor response Object manipulation Localization to noxious stimulation Flexion withdrawal Abnormal posturing None	Command responsivity Response to command (open/close eyes, mouth,move finger, hand,leg)	− 0.66	< 0.00001
	Threat Eye blink		
	Tactile Head or eye orientation or shoulder movement to tap		
	Tactile Withdrawal or eye blink or mouth twitch		
	Pain Withdrawl or other response linked to stimulus		
	Pain Withdrawal or other response linked to stimulus		
Oral-verbal motor Intelligible verbalization Vocalization oral movement Oral reflexive movement None	Vocalization Spontaneous word; Non verbal vocalization	− 0.53	< 0.00001

CNCS items within each domain were summed per subject, where appropriate and as shown

To examine all pairs of CRS-R and CNCS assessments, we applied *repeated measures correlation,* [25, 26]. We analysed the correlation between pairs of CRS-R and CNCS overall assessments, while accounting for multiple within-subject assessments, which in our study were performed at subsequent timepoints variable in number (Supplementary Method 1). Then, items of the CNCS scale were grouped by sensorimotor domain (shown in Table 1). Spearman’s correlation was calculated between CRS-R and CNCS domain scores to assess the evaluation concordance of the two scales in each domain.

We charted the median values scored in each CRS-R domain by patients located at different levels of the CNCS scale, based on all pairs of assessments (Supplementary Fig. 2 top); and we plotted the median scores received at each CNCS item by patients in the low (0 to 7), medium (8 to 16), and high (≥ 17) bands of CRS-R, thus spanning the full range of possible scores (Supplementary Fig. 2 bottom).

#### Scale concordance with the clinical status

We assessed the concordance of the patients’ clinical status with the scores obtained at the CRS-R. Based on the theoretical framework described above (also in supplementary Table 1), each CRS-R evaluation was associated with the corresponding clinical status. The distribution of the corresponding CNCS levels was then computed for each CRS-R-based clinical status, and the results are reported in the concordance matrix in Fig. 2 left.

Analogously each CNCS evaluation was associated with the corresponding clinical status, and the correspondence with the CRS-R-based clinical status assessed (con-fusion matrix in Fig. 2 right). Theoretical agreement is achieved for the assessment pairs located on the main diagonal of the matrix.

#### Patients’ level of consciousness and clinical status over time and 1 year outcome

Patients’ level of consciousness was evaluated with both CRS-R and CNCS scales. Scores were charted for all available timepoints at 3, 6, 9 and 12 months (Fig. 3a, b). Unavailable timepoints were extracted retrospectively from the patients’ records if possible (missing timepoints = 5). Of the 40 patients included, 37 were followed up for longer than 1 year from the event, and 3 were lost. Domains of behavioral change over time were studied from all consecutive assessments and from both scales. This analysis is detailed in Supplementary Material 3.

The clinical outcome (coma, VS, MCS −/ + , eMCS) at 1 year follow-up was determined through clinical examination, CRS-R, and CNCS scores, and is presented in Fig. 4.

## Results

### Patients

Ninety-seven patients with a diagnosis of acquired brain lesion of traumatic, anoxic, vascular or infectious etiology and with a clinical diagnosis of UWS or MCS were admitted in the 2015–2020 period to our Scientific Institute, where they received a clinical–functional assessment and rehabilitation interventions.

Twenty-seven patients were excluded, because they were younger than 5 years at first assessment; 16 were older than 18 years at first assessment; 2 had a previous diagnosis; 9 had time > 12 months between the ABI and the first assessment; 3 had cerebral tumor.

Forty patients (28 males; 12 females) met the inclusion criteria and entered the study. The majority of patients were white (92.5%), 5% were mixed and 2.5% were asian. Twenty-one patients of 40 (52.5% of the total sample) reported a traumatic brain injury (TBI), 6 (15%) had anoxia, 10 (25%) vascular brain injury, and 3 (7.5%) severe encephalitis due to infection. Two groups were established: patients who reported a TBI entered the “traumatic” group (*N* = 21), and those who suffered a brain lesion in consequence of any other etiology formed the “non-traumatic” group (*N* = 19). Table 2 describes the clinical and demographic characteristics of the total sample, “traumatic”, and “non-traumatic” groups.

**Table 2:** Demographic characteristics, severity at event (GCS score), outcome at admission to the rehabilitation center (GOS-E score), clinical characteristics during in-stay and administration of previous rehabilitation (i.e., prior to admission to our center)

	Total sample (*n* = 40)		Traumatic (*n* = 21)		Non traumatic (*n* = 19)		Statistics
	Mean	SD	Mean	SD	Mean	SD	*t* Test
Age at injury (months)	133.98	43.25	147.81	46.104	118.68	34.95	0.053
Age at first assessment (months)	137.05	43.76	150.81	47.030	121.84	35.03	0.046
	Median	Range	Median	Range	Median	Range	Mann Whitney test
GCS score	4	3–12	4	3–7	5	3–12	0.238
GOS E-score	2	2–3	2	2–3	2	2–3	0.837
	*n*	%	*n*	%	*n*	%	*X*^2^
Gender							
Male	28	70.0	13	61.9	15	79.0	0.240
Female	12	30.0	8	38.1	4	21.0	
Need for neurosurgery	26	66.7	12	57.1	14	73.7	0.273
Tracheotomy	25	64.1	16	76.2	9	47.4	0.060
Feeding disorders							
Absence of disorders	3	7.7	2	9.5	1	5.3	0.689
Dysphagia	1	2.5	1	4.8	0	0.0	
Nasogastric tube (NGT)	5	12.5	3	14.3	2	10.5	
Percutaneous endoscopic gastrostomy (PEG)	31	77.5	15	71.4	16	84.2	
Paroxysmal sympathetic hyperactivity episodes	12	30.8	7	33.3	5	26.3	0.629
Motor impairment							
Absence of impairment	1	2.5	1	4.8	0	0.0	0.259
Motor retardation	2	5.0	2	9.5	0	0.0	
Hemiparesis	1	2.5	1	4.8	0	0.0	
Quadriparesis	36	90.0	17	81.0	19	100.0	
Previous rehabilitation	3	7.7	2	9.5	1	5.3	0.609

Data are presented for the overall sample, and for the subgroups who had either traumatic or non-traumatic type of brain injury

The mean age at injury was 134 months (traumatic: 148 months, non-traumatic: 119 months), the mean GCS score at rescue was 4 (traumatic: 4, non traumatic: 5). Median time between event and admission to our rehabilitation center was 56 days (IQR 39–77). The median time to first responsivity was 89 days with CRS-R assessment (IQR 59–157; first starkey in any domain), and 67,5 days with CNCS assessment (IQR 54–96, first score to zero in any scale), for patients who emerged from UWS. The median time to follow commands was 90 days with CRS-R assessment (IQR 63–147, score 3 or 4 at auditory scale); and 89 days with CNCS assessment (IQR 63–138,5, score zero at item 2). The mean GOS-E score at admission was 2 (traumatic: 2, non-traumatic: 2).

The total number of CRS-R assessments was 196, which corresponds to an average of 4,9 assessments per patient, during 72 days in median (IQR 31–168). Correspondingly, the total number of CNCS assessments was 212 (average: 5,3 assessments per patient, over 104 days in median; IQR 30–167). Median time from injury to first CRS-R assessment was 70 days (IQR 54–87); median time from injury to first CNCS administration was 68 days (IQR 50–89). The total combinations of CRS-R and CNCS assessments, performed within a 5 days time window, were 189 (average per patient: 4,7).

### Characterization of the CRS-R and CNCS instruments

GRM analysis of the CRS-R showed that the *motor function* domain captured the largest discrimination difference, both in general and at earliest assessment. *The visual* and *auditory functions* were the next most important domains (Supplementary Material 5). The CRS-R test information function revealed a bimodal distribution located around − 1.5 and + 1.5 theta, initially led by the *motor function*, and by the *auditory function* at later assessments. The test characteristic curve showed dependency on aetiology, with lower expected scores for non-traumatic cases.

Analysis of the CNCS showed much more calibrated test characteristic curve overall, with the *visual* items conveying the largest discrimination difference, and unimodal distribution of the test information function centered around null theta. CNCS level showed lower discrimination power compared to the use of single items. The test characteristic curve revealed no dependency on aetiology.

### Correlation between the CRS-R and the CNCS scales

**Fig. 1:**
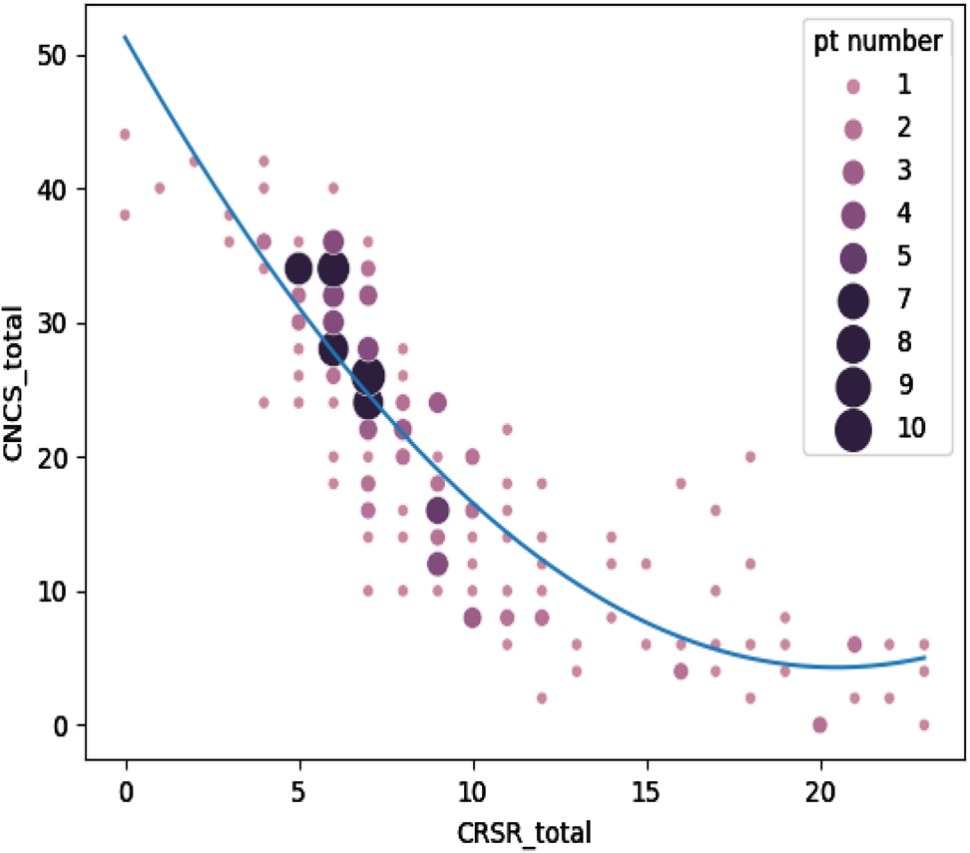
Pairs of CRS-R and CNCS assessments. The CRS-R total score (*x* axis) is plotted against the CNCS total score (*y* axis). Overall, high end moderate correlation was observed between the two assessment instruments, and a non-linear trend (blue line). Each combination of CRS-R and CNCS total scores is indicated by a pink dot. Dots bigger in size and darker in shade indicate that the score combination was obtained for more than one evaluation in the dataset

The examination of the CRS-R and CNCS assessment pairs showed overall high end moderate correlation between the two scales (Fig. 1), as rated by *Chan criteria* for medical correlations, and a non-linear trend (blue line). Specifically, the lowest CNCS scores (0–8) approximately matched scores 13–23 of the CRS-R. For these scores, which likely correspond to the highest levels of consciousness measured by the two scales, CNCS demonstrated less discriminatory ability compared to the CRS-R, which indeed provided a more fine-grained description of the patients’ status. Quantitatively, repeated measures correlation coefficient between pairs of CRS-R and CNCS assessments was *r* = − 0.713 (*p* = 2.73e-24, CI95%: − 0.78, − 0.62), indicating highly significant inverse correlation between the two scales CRS-R and CNCS, after adjustment for inter-individual variability (Supplementary Fig. 1).

### Concordance between the CRS-R and CNCS scores

The analysis of the CRS-R domain scores confirmed that CRS-R scores in the visual domain increased when CNCS levels decreased (i.e., when patients improved from coma towards emergence to consciousness, or were less severe). This was also observed for the CRS-R auditory domain, which however scored 1 for both levels 4 and 3 of the CNCS. Patients in CNCS levels 4, 3 and 2 all scored 2 in median at the CRS-R motor domain, which seems to best track later improvements, at levels 1 and 0. CRS-R oral-verbal domain appeared insensitive to changes tracked by the CNCS levels, and the CRS-R communication domain only tracked changes occurring at emergence to consciousness in our pediatric cohort (Supplementary Fig. 2 top).

The analysis of CNCS items showed that responses to *pain* (i.e., items 9 and 10) were already present in median in patients scoring low (i.e., 0 to 7) at the CRS-R; while *command responsivity*, *visual, threat, olfactory, and tactile* responses, and *vocalisation* (i.e., items 2 to 7 and item 11) were absent. In patients scoring mid-range at the CRS-R (i.e., 8 to 16), the *auditory*, *visual, threat and tactile* responses (i.e., items 1, 4, 5, 8) were also present on average. Patients scoring high (i.e., 17 to 23) at the CRS-R still overall failed to provide robust *olfactory* and *vocalisation* responses (Supplementary Fig. 2 bottom). The comprehensive list of CNCS items is reported in Supplementary material 4.

### Concordance between the CRS-R and CNCS scores, and the clinical status

**Fig. 2:**
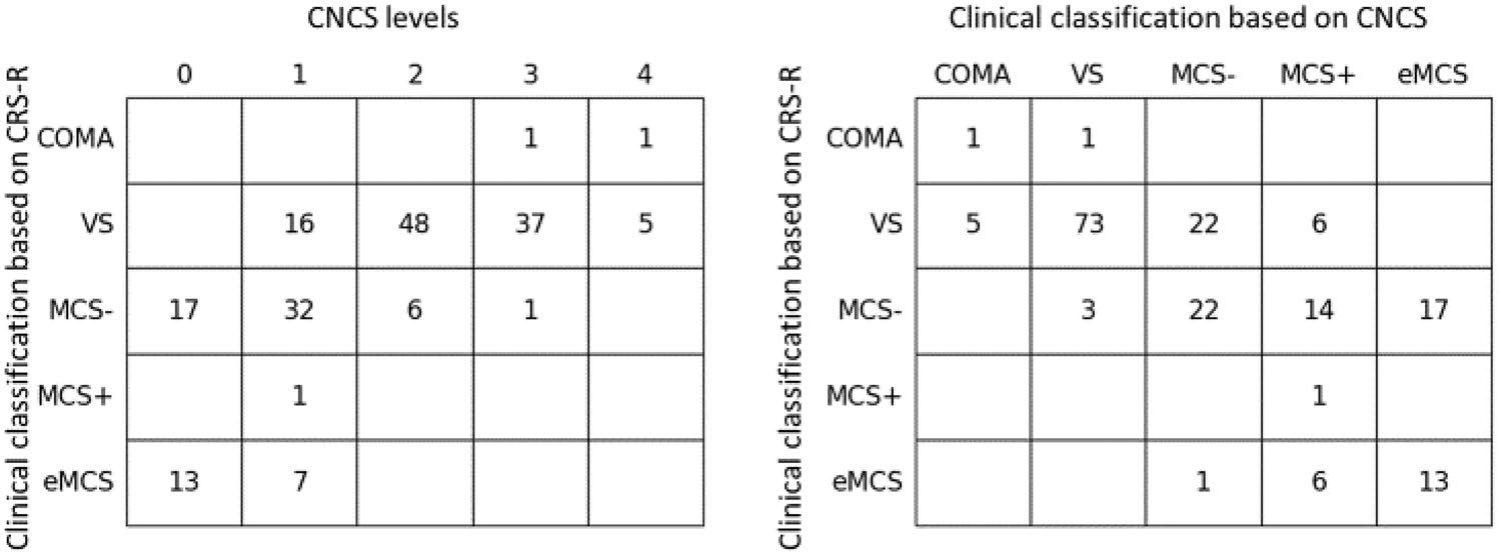
(Left) Relation between CRS-R scores, grouped by corresponding clinical condition, and CNCS levels. (Right) Relation between CRS-R and CNCS scores, grouped by corresponding clinical condition. Data is shown for all the pairs of CRS-R and CNCS assessments. *VS* vegetative state, *MCS* minimally conscious state minus, *MCS + * minimally conscious state plus, *eMCS* emergence to consciousness

We matched the CRS-R scores to the clinical status, according to the framework in the Supplementary material 3. VS, defined according to the CRS-R, mainly corresponded to CNCS levels 2 and 3. MCS- predominantly corresponded to CNCS level 1, and emergence from MCS to CNCS 0 (Fig. 2).

Then, we similarly matched the CNCS scores to the clinical status, and we obtained full clinical status concordance in 110 of 185 cases (59%) between the two scales. Among the most severe mistakes (off-diagonal cases), 17 patients were in MCS- according to CRS-R and eMCS based on CNCS; and one patient was in eMCS according to CRS-R, and in MCS-based on CNCS.

### Evolution of the level of consciousness over time, and overall clinical outcome at 1 year

**Fig. 3:**
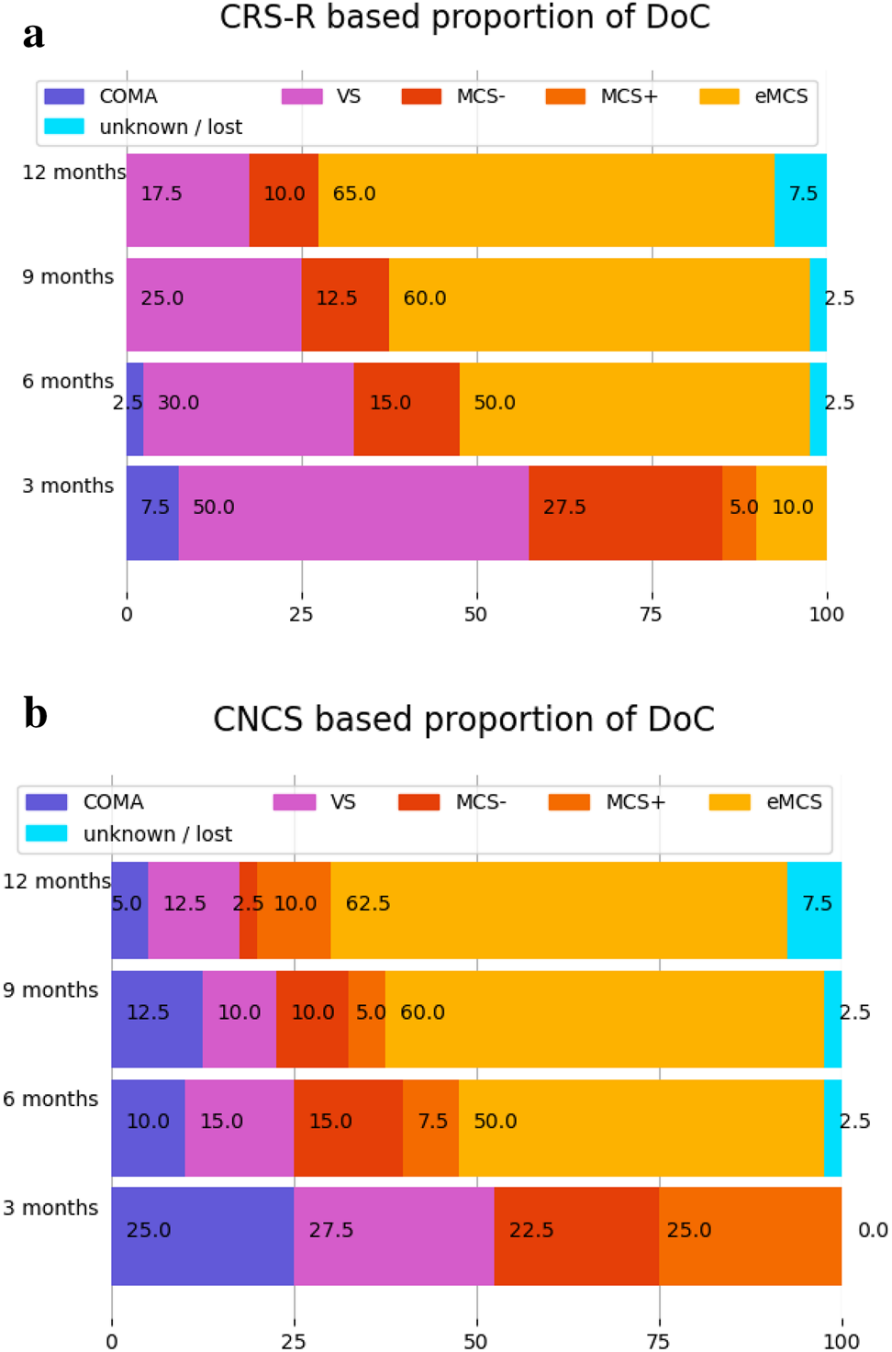
**a**, **b** Patients’ level of consciousness at 3, 6, 9, 12 months after event. Data is expressed in percentage (*n *= 40). *VS* vegetative state, *MCS− / + * minimally conscious state minus/plus, *eMCS* emergence to consciousness

We analysed the level of consciousness assessed by CRS-R and CNCS at 3, 6, 9 and 12 months after event (Fig. 3a, b). Based on CRS-R, at 3 months, 3 (7.5%) of 40 patients were still in a coma, half were in UWS, and only 4 (10%) had emerged to consciousness. At 6 months, patients in UWS had reduced to 12 (30%), while half patients had emerged. At 9 months, most (*n* = 24, 60%) of patients had emerged, with 10 (25%) still in UWS, and none in a coma anymore. At 12 months, 26 (65.0%) had emerged.

**Fig. 4:**
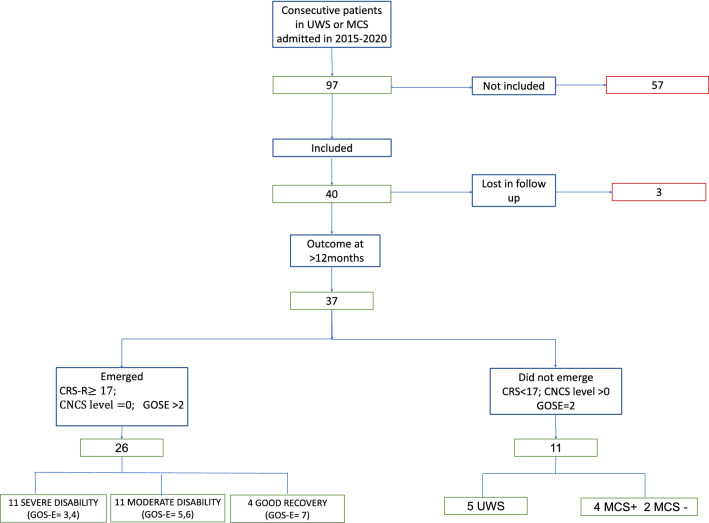
Flowchart showing patients’ enrollment and overall clinical outcome 12 months after brain injury

We also considered the clinical outcome at 1 year after brain injury, which was available for 37 (92.5%) of 40 patients. Of these 37 patients, 26 (70.2%) emerged to consciousness with CRS-R > 17, CNCS = 0, GOS-E > 2, or recorded clinical observations indicating emergence. The clinical outcome of the remaining 11 (29.7%) of 37 indicated they had not (yet) emerged to consciousness. Of these patients, 5 were observed to be in UWS, 2 in MCS-, and 4 MCS + (Fig. 4) based on the joint information provided by CRS-R, CNCS, GOS-E, and clinical observations. Of note, in four cases, the clinical outcome at 1 year after brain injury did not correspond with the CRS-R classification alone (status at 12 months in Fig. 3 vs. Fig. 4).

## Discussion

We administered the Coma Recovery Scale revised (CRS-R) to a cohort of 40 children and adolescents (5–18 years) with a Disorder of Consciousness, who initially presented in UWS/VS or MCS. The CRS-R is the gold standard neurobehavioral instrument for the assessment of DoC in adults, [7]. A validation on pediatric patients is needed, to grant diagnostic and prognostic comparability with the scientific literature on adult cohorts, [9]. In this study, we compared neurobehavioral assessments using CRS-R to those obtained through the Coma Near Coma Scale (CNCS), previously studied in children, [19] and used here as the standard instrument.

Concordant with previous observations on healthy children, [21] that highlight the importance of using a modified version of the CRS-R in children younger than 59 months, we based our work on a cohort of children aged 5 years and older.

The CRS-R showed overall moderate correlation with the CNCS (*r* = − 0.71) when applied within a 5 days timeframe, indicating good agreement and expected opposite direction, with highest CRS-R values and lowest CNCS scores indicating mild condition. Similarly, previous work by our group found *fair agreement* between CNCS and the LOCFAS, [19].

Beyond overall correlation, areas of score magnification in one scale with respect to the other (i.e., horizontal and vertical segments of the correlation line, such as the one at bottom right of Fig. 1) suggest floor effects. Specifically, there is indication that subjects’ CRS-R overall score can increase significantly at relatively stable values of CNCS.

The analysis of single CRS-R domains showed that the decrease in CNCS levels corresponded to gradual score increase in the visual and auditory domains at the CRS-R, indicating close mapping in these domains by the two instruments. Conversely, the dynamics of the motor domain responses probed by the CRS-R was captured by the lowest CNCS levels only (i.e., CNCS levels 0 to 2; milder condition), probably reflecting the pivotal role of motor responses in discerning the emergence from UWS/VS or above [27].

Likewise, GRM showed that the discriminative power of the CRS-R instrument relies primarily on the *motor abilities*, followed by the *visual* and *auditory* functions, and that its informative characteristics follow a bimodal distribution in two distinct ability ranges. Of note, auditory items in the CRS-R imply some degree of motor abilities. Conversely, the CNCS mostly relies on *visual* functions.

Overall, our data suggest that the assessment of the visual and auditory domains through CRS-R holds discriminative power along the entire spectrum of DoC in children. Conversely, the motor domain assessment is most informative to refine the diagnosis when minimal signs of consciousness have already been detected in other domains. The request of more structured and/or refined motor and auditory responses in CRS-R (compared with CNCS) might be at the origin of its better discriminative power towards the milder DoC, when higher neuromotor and neurocognitive demands can be met by the patient. However, this may also indicate that only a sub-group of patients can access the motor requests of CRS-R, with the most severe cases left outside the scale sensitivity in the motor domain, due to the difficulty of the requests. Also, in our cohort, patients with extreme motor impairment, such as severe spastic tetraplegia, and those with specific oral-motor impairment hardly accessed items in the CRS-R motor and oral verbal domains, as too demanding. Lack of performance in the motor and oral verbal domains resulted in low CRS-R total score. Importantly, this might lead to potential underestimation of the patients’ real cognitive abilities and overall level of consciousness. In these cases, a minimal consciousness state or even emergence to consciousness could be clinically observed, which remained undetected by the scale. Reportedly, these children appeared to understand the request, but either failed to accomplish the task or the execution was judged highly inconsistent by the clinician.

These observations have one important implication in clinics. Suspicion of covert consciousness or cognitive-motor dissociation could question the isolated use of CRS-R for assessment, and favour the combined use of scales based on multiple unimodal sensory requests such as CNCS and LOCFAS. The CNCS demonstrated higher measurement robustness in these cases, probably due to the easiness of the motor request and the limitedness of motor assessment (i.e., one item only: *response to command*); and it also anticipated of a few days the emergence from VS/UWS to MCS-, compared to the CRS-R.

Few studies described the outcomes of children in DoC who received rehabilitation; and even fewer examined the changes and outcome at very long-term (i.e., > 1 year post severe brain injury), [4, 28]. We were able to measure the outcome of 37 patients, 12 months past the event. Our results show that patients’ clinical status gloabally improved throughout the first year after event, with 2 out of 3 patients emerged to consciousness after 1 year. Eleven patients only did not emerge (5 in UWS, 2 in MCS−, and 4 MCS + , if considering the overall clinical outcome). Also, we show that emergence can occur several months after the event, and should not be considered an exceptional event, at least until 1 year past the brain injury.

One major theme in the assessment of pediatric DoC is the personalization of the stimuli. Recent literature agrees on the importance of *saliency* (i.e., emotional or affective relevance) in the stimulus choice and administration to maximise arousal and achieve the best patient’s responsiveness, [29–32]. In our center, a personalised strategy is adopted for rehabilitation and clinical assessment; the preferred colors, tunes, and familiar voices are embedded in the stimuli choice and delivery for each patient, based on a pre-assessment of the patients’ interests, hobbies and preferences during the family/caregivers interview. This strategy was not implemented during the administration of both CRS-R and CNCS scales, to avoid inconsistencies in the administration protocol.

For some children of this cohort, affective reactions to salient stimuli were observed during clinical evaluations, in the absence of any other response. Previous literature has termed this evidence *contingent affect,* [5, 33]. This often manifested as smiles in social contexts with highly arousing circumstances, such as the parent’s embrace, kisses and cuddles. We observed these affective reactions at low levels of consciousness, including MCS−.

Our study raises methodological considerations. By design, neither CNCS levels, nor CNCS scores at single items, map specific levels of consciousness. CRS-R and CNCS instruments have inherently different structure: CRS-R is organised in ordinal subdomains, while CNCS is made up of independent items. Also, despite probing largely similar cognitive and motor abilities, the two scales assess abilities differently, with CRS-R giving larger emphasis to the motor domain. For all these reasons, the comparison of CRS-R and CNCS on the common ground of the levels of consciousness is challenging, probably inaccurate, and most likely causes the off-diagonal mismatches in Fig. 2.

We also acknowledge that our work has some limitations. As the two instruments, CRS-R and CNCS, are intended to measure the same construct (i.e., responsiveness to stimuli), demonstration of correlation is a rather weak validation, and for this reason multiple analysis approaches were pursued. Also, our attempt to map each item/domain score to the levels of consciousness might have injected arbitrarity and/or DoC level misassignments. Time from injury to study enrollment was highly inhomogeneous in our cohort. Also, patients were followed-up for different durations. However, this contributed to capture all the stages of the DoC disease from post-acute to late chronic overall. Also, our study does not address the topic of DoC assessment in toddlers and newborns, as we investigated ages older than 5 years. Likewise, we were unable to investigate whether mismatches in the assignment of the level of consciousness would have similar rates in adult cohorts when administering CRS-R and CNCS, as we cannot recruit adult patients in our pediatric center.

## Conclusions

The CRS-R is the gold standard neurobehavioral instrument for the assessment of DoC, designed to be used with adult cases. We show that CRS-R can track changes in DoC in children as young as 5 years old, and we provide evidence that the agreement with CNCS scores is good, particularly in the visual and auditory domains. We observe that the motor and communication requests of CRS-R are more demanding than what is required by CNCS. Thus, unsurprisingly CRS-R has higher sensitivity in tracking refined motor and communication abilities typical of the emergence from MCS + towards severe disability (and normal consciousness). This results in a penalising effect of CRS-R towards scoring DoC in presence of severe motor impairment and probably cognitive-motor dissociation, when the diagnosis is typically most difficult. This last diagnostic domain warrants further investigation.

## Supplementary Information

Below is the link to the electronic supplementary material.

Supplementary file1 (DOCX 109328 KB)
