# Supplementary material for: Neuropsychological assessment through Coma Recovery Scale-Revised and Coma/Near Coma Scale in a sample of pediatric patients with disorder of consciousness
Source: J Neurol. 2022 Nov 5;270(2):1019–29. doi: 10.1007/s00415-022-11456-6 (PMC9886605; doi:10.1007/s00415-022-11456-6)
Supplement: Supplementary file 1 — Supplementary file1 (DOCX 109328 KB) [file 415_2022_11456_MOESM1_ESM.docx]

**SUPPLEMENTARY MATERIAL**

**Neuropsychological assessment through Coma Recovery Scale Revised and Coma Near Coma Scale in a sample of pediatric patients with disorder of consciousness**

**Supplementary Table 1**. Framework for theoretical association between clinical status and CNCS and CRS-R levels. A third column, also associating the Level of Consciousness Functional Assessment Scale, is reported for convenience, and discussed in previous work [4]. MCS: minimally conscious state. CRS-R: Coma Recovery Scale Revised; CNCS: Rappaport Coma/Near Coma Scale; LOCFAS: Level of Cognitive Functioning Assessment Scale.

| Clinical status | CRS-R criteria | CNCS Level | CNCS additional criteria | LOCFAS level | LOCFAS additional criteria |
| --- | --- | --- | --- | --- | --- |
| Emerge | ((Motor domain =6) OR  (Communication domain =2)) | Level 0 | - | Level 5 | |
|  |  |  |  | Level 4 | |
| MCS+ | ((Auditory domain =4) OR (Visual domain =5) OR (Oral-verbal domain =3))  AND  Motor domain <6  AND  Communication domain <2 | Level 1 | And ‘Command responsivity’ is 0 | Level 3 | If specific score at item ‘Execution of commands’ is 2, 3 or 4 |
| MCS- | (Auditory domain =3) OR  (Visual domain =2 to 4) OR  (Motor domain  =3 to 5) OR (Communication domain =1)  AND  Oral-verbal domain =2  AND  Arousal >0 | Level 1 | And ‘Command responsivity’ is 2 or 4 | Level 3 | If specific score at item ‘Execution of commands’ is 1 |
|  |  | Level 2 | If ‘Auditory’ is 0,  or if ‘Visual (item n.3 or 4)’ is 0, or both |  |  |
| Vegetative state | ((Auditory domain =1 or =2) OR  (Visual domain =1) OR  (Motor domain =2) OR  (Oral-verbal domain =2))  AND  Communication domain =0  AND  (Arousal =1 or =2) | Level 2 | If ‘Auditory’ is not 0  And ‘Visual (item n.3 or 4)’ is not 0 | Level 2 |  |
|  |  | Level 3 |  |  |  |
| Coma | If not satisfying criteria for higher levels | Level 4 |  | Level 1 |  |

**Supplementary method 1**. *Repeated measures correlation* is a statistical technique for determining the common within-individual association for paired measures assessed on two or more occasions for multiple individuals (Bakdash & Marusich, 2017). It accounts for non-independence among observations using analysis of covariance (ANCOVA) to statistically adjust for inter-individual variability. By removing measured variance between-participants, repeated measures correlation provides the best linear fit for each participant using parallel regression lines (the same slope) with varying intercepts. Like a Pearson correlation coefficient, the coefficient is bounded by − 1 to 1 and represents the strength of the linear association between two variables. Two-tailed rm_corr implementation in pingouin0.3.10 was used (Vallat, 2018).

**Supplementary Figure 1**. Repeated measures correlation plot. Each dot represents a pair of CRS-R and CNCS assessments. Dots depicted in the same color represent within-subject pairs of assessments at different timepoints. Each line represents the best linear fit for each subject, after correction for inter-subject variability. Intercept thus varies between subjects.

**Supplementary Figure 2** (Top) Median (and IQR) scores in each CRS-R domain corresponding to each CNCS level. (Bottom) Median (and IQR) scores for each CNCS item corresponding to ranges of CRS-R values (namely, from 0 to 7, from 8 to 16, and 17 or higher)

**Supplementary Massterial 3**. *Domains of behavioral change.* For each patient, all available couples of CNCS and CRS-R assessments were considered. When consecutive assessments were also performed at different timepoints, data was considered for the analysis of within-subject behavioral changes. For each couple of consecutive timepoints, earlier and later CNCS levels were used to chart the corresponding changes in each CRS-R domain. In each domain, a positive change (improvement) was counted if the domain score increased by one point or more. Correspondingly, a negative change was counted if the domain score decreased by one point or more. Results were normalised per the number of subjects transitioning between the same two early-to-late CNCS levels, and imaged (Supplementary Figures below). Early-to-late CNCS level combinations relying on less subjects provide less robust results. This analysis is descriptive, as powered statistical analysis would require a much larger sample size. Computations and graphical representation were conducted in Python 3.8.5.

**Supplementary Figure 3 - Analysis of positive behavioral change** (“improvement”). All consecutive assessments performed at different timepoints, and for all patients, were considered. Each panel depicts the transition/changes in levels of consciousness from earlier (x-axis) to later (y-axis) CNCS levels, and include: overall improvements at CNCS (i.e., top left triangle), no change (i.e., on diagonals), and overall worsening (i.e., bottom right triangle). Positive changes in each CRS-R domain are charted (coloured squares) for the transition from earlier (x-axis) to later (y-axis) CNCS levels. A positive change in any CRS-R domain was considered a CRS-R domain improvement. CRS-R domain improvements were counted if the domain score increased by one point or more. Results were normalised per number of subjects transitioning between the same two early-to-late CNCS scale levels, as indicated in the bottom right plot. The pseudocolor bar indicates normalised values (1=all cases improved at CRS-R domain, for specific level change in CNCS; 0=no cases improved at CRS-R domain, for specific level change in CNCS). Numbers inside the bottom right panel indicate the total count of assessment pairs available for each x-y CNCS combination and both instruments (CRS-R and CNCS), and constitute the denominator number of the normalisation at each CNCS level combination (x-y).

**Supplementary Figure 4 -** **Analysis of negative behavioral change** (“worsening”). All consecutive assessments were considered in pairs. Negative changes in each CRS-R domain are charted for the transition from earlier (x-axis) to later (y-axis) CNCS levels. A negative change in any CRS-R domain was considered as worsening. Worsening events were counted if the domain score decreased by one point or more. Results were normalised. Number of assessment pairs are distributed as per the previous image (bottom right panel).

**Supplementary Material 4**. Description of CNCS items

| item | Individual Parameters | Stimulus |
| --- | --- | --- |
| 1 | Auditory | Bell ringing 5 seconds at 10 seconds intervals |
| 2 | Command Responsivity | Request patients to open or close eyes, mouth, or move finger, hand or leg |
| 3 | Visual | Light flashes (1/sec x 5) in front, slightly left, right and up and down each trial |
| 4 | Visual | Tell patient” Look at me”; move face 20” away, from side to side |
| 5 | Threat | Quickly move hand forward to within 1”-3” of eyes |
| 6 | Olfactory | Ammonia capsule/bottle 1” under nose for about 2 seconds |
| 7 | Tactile | Shoulder tap- tap shoulder briskly 3X without speaking to patient; each side |
| 8 | Tactile | Nasal swab (each nostril) |
| 9 | Pain | Firm pinch on finger tip; pressure of wood pencil across nail; each side |
| 10 | Pain | Robust ear pinch/pull 3X; each side |
| 11 | Vocalisation | None. score best response |

**Supplementary Material 5.** Item response theory (IRT) analysis of CRS-R and CNCS scales

**Item response theory (IRT) analysis of CRS-R scale**

**IRT Model – Graded response model for first CRS-R assessment**

Results are sorted by descending discrimination power.

Graded response model

Number of observations = 40

Iterations to convergence = 11

Log likelihood = -176.34

|  | Coefficient | Std. err. | Z | P>\|z\| | [95% confidence interval] | |
| --- | --- | --- | --- | --- | --- | --- |
| **Motor Function** | | | | | | |
| Discrim. difference | 11.94 | 10.34 | 1.16 | 0.248 | -8.32 | 32.20 |
| >=1 | -1.24 | 0.32 |  |  | -1.87 | -0.60 |
| >=2 | -1.08 | 0.28 |  |  | -1.64 | -0.52 |
| >=4 | 0.41 | 0.22 |  |  | -0.02 | 0.84 |
| >=5 | 0.65 | 0.24 |  |  | 0.17 | 1.13 |
| =6 | 0.88 | 0.28 |  |  | 0.34 | 1.43 |
| **Visual function** | | | | | | |
| Discrim. difference | 7.92 | 3.50 | 2.26 | 0.024 | 1.07 | 14.8 |
| >=1 | -0.51 | 0.19 |  |  | -0.89 | -0.13 |
| >=2 | 0.35 | 0.20 |  |  | -0.04 | 0.75 |
| >=3 | 0.54 | 0.23 |  |  | 0.09 | 0.98 |
| >=4 | 0.74 | 0.26 |  |  | 0.24 | 1.24 |
| =5 | 1.29 | 0.33 |  |  | 0.64 | 1.94 |
| **Auditory function** | | | | | | |
| Discrim. difference | 5.89 | 2.09 | 2.82 | 0.005 | 1.80 | 9.98 |
| >=1 | -0.94 | 0.25 |  |  | -1.44 | -0.45 |
| >=2 | 0.48 | 0.22 |  |  | 0.04 | 0.91 |
| >=3 | 0.64 | 0.24 |  |  | 0.17 | 1.11 |
| =4 | 1.30 | 0.35 |  |  | 0.62 | 1.98 |
| **Communication scale** | | | | | | |
| Discrim. difference | 2.85 | 1.09 | 2.62 | 0.009 | 0.72 | 4.98 |
| >=1 | 0.97 | 0.33 |  |  | 0.33 | 1.62 |
| =2 | 2.09 | 0.52 |  |  | 1.06 | 3.12 |
| **Oral-verbal function** | | | | | | |
| Discrim. difference | 2.61 | 0.83 | 3.16 | 0.002 | 0.99 | 4.23 |
| >=1 | -1.88 | 0.51 |  |  | -2.89 | -0.88 |
| >=2 | 0.60 | 0.28 |  |  | 0.06 | 1.14 |
| =3 | 2.61 | 0.65 |  |  | 1.33 | 3.89 |
| **Awareness scale** | | | | | | |
| Discrim. difference | 2.20 | 0.67 | 3.29 | 0.001 | 0.89 | 3.50 |
| >=1 | -2.06 | 0.58 |  |  | -3.19 | -0.93 |
| >=2 | -0.36 | 0.24 |  |  | -0.84 | 0.12 |
| =3 | 1.62 | 0.47 |  |  | 0.70 | 2.53 |

**IRT Model – Graded response model for all CRS-R assessments**

Results are sorted by descending discrimination power.

Graded response model

Number of observations = 196

Iterations to convergence = 5

Log likelihood = -912.06

|  | Coefficient | Std. err. | Z | P>\|z\| | [95% confidence interval] | |
| --- | --- | --- | --- | --- | --- | --- |
| **Motor Function** | | | | | | |
| Discrim. difference | 5.05 | 1.00 | 5.07 | <0.001 | 3.09 | 7.00 |
| >=1 | -2.02 | 0.24 |  |  | -2.48 | -1.56 |
| >=2 | -1.56 | 0.16 |  |  | -1.86 | -1.25 |
| >=3 | 0.70 | 0.11 |  |  | 0.49 | 0.91 |
| >=4 | 0.72 | 0.11 |  |  | 0.51 | 0.93 |
| >=5 | 1.02 | 0.12 |  |  | 0.78 | 1.26 |
| =6 | 1.40 | 0.14 |  |  | 1.12 | 1.69 |
| **Auditory function** | | | | | | |
| Discrim. difference | 4.61 | 0.82 | 5.63 | <0.001 | 3.00 | 6.21 |
| >=1 | -1.36 | 0.14 |  |  | -1.64 | -1.08 |
| >=2 | 0.25 | 0.09 |  |  | 0.06 | 0.43 |
| >=3 | 0.98 | 0.12 |  |  | 0.75 | 1.22 |
| =4 | 1.51 | 0.15 |  |  | 1.21 | 1.80 |
| **Visual function** | | | | | | |
| Discrim. difference | 3.64 | 0.54 | 6.79 | <0.001 | 2.59 | 4.69 |
| >=1 | -0.46 | 0.10 |  |  | -0.66 | -0.27 |
| >=2 | 0.25 | 0.10 |  |  | 0.06 | 0.44 |
| >=3 | 0.46 | 0.10 |  |  | 0.26 | 0.66 |
| >=4 | 1.21 | 0.14 |  |  | 0.94 | 1.48 |
| =5 | 1.62 | 0.17 |  |  | 1.30 | 1.95 |
| **Communication scale** | | | | | | |
| Discrim. difference | 3.03 | 0.58 | 5.26 | <0.001 | 1.90 | 4.15 |
| >=1 | 1.22 | 0.15 |  |  | 0.92 | 1.51 |
| =2 | 2.17 | 0.24 |  |  | 1.70 | 2.63 |
| **Awareness scale** | | | | | | |
| Discrim. difference | 1.98 | 0.31 | 6.46 | <0.001 | 1.38 | 2.59 |
| >=1 | -2.73 | 0.39 |  |  | -3.50 | -1.96 |
| >=2 | -0.94 | 0.15 |  |  | -1.22 | -0.65 |
| =3 | 2.13 | 0.26 |  |  | 1.63 | 2.63 |
| **Oral-verbal function** | | | | | | |
| Discrim. difference | 1.64 | 0.25 | 6.54 | <0.001 | 1.15 | 2.13 |
| >=1 | -2.87 | 0.42 |  |  | -3.70 | -2.04 |
| >=2 | 0.42 | 0.14 |  |  | 0.15 | 0.69 |
| =3 | 2.74 | 0.38 |  |  | 2.00 | 3.48 |

**Boundary characteristic curves for CRS-R subscales**

First assessment	All assessments

**Category characteristic curves for CRS-R subscales**

First assessment	All assessments

**Test characteristic curves for CRS-R**

First assessment	All assessments

**Test Information functions for CRS-R**

First assessment	All assessments

**Item Information functions for CRS-R subscales**

First assessment	All assessments

**Item response theory (IRT) analysis of CNCS scale**

**IRT Model – Graded response model for first CNCS assessment**

Results are sorted by descending discrimination power.

Graded response model

Number of observations = 40

Iterations to convergence = 6

Log likelihood = -319.89

|  | Coefficient | Std. err. | Z | P>\|z\| | [95% confidence interval] | |
| --- | --- | --- | --- | --- | --- | --- |
| **Item 4 – Visual – Fixation & tracking** | | | | | | |
| Discrim. difference | 5.50 | 2.50 | 2.20 | 0.028 | 0.60 | 10.40 |
| >=2 | -0.60 | 0.23 |  |  | -1.05 | -0.15 |
| =4 | 0.01 | 0.19 |  |  | -0.37 | 0.38 |
| **Item 3 – Visual – Fixation or avoidance** | | | | | | |
| Discrim. difference | 5.22 | 2.16 | 2.41 | 0.016 | 0.98 | 9.46 |
| >=2 | -0.53 | 0.22 |  |  | -0.97 | -0.09 |
| =4 | -0.18 | 0.20 |  |  | -0.57 | 0.20 |
| **Item 2 – Command responsivity** | | | | | | |
| Discrim. difference | 5.10 | 2.21 | 2.31 | 0.021 | 0.77 | 9.42 |
| >=2 | -0.37 | 0.21 |  |  | -0.79 | -0.05 |
| =4 | 0.00 | 0.19 |  |  | -0.37 | -0.37 |
| **Item 9 – Pain – Firm pinch finger tip-** | | | | | | |
| Discrim. difference | 2.74 | 1.14 | 2.41 | 0.016 | 0.51 | 4.98 |
| >=2 | 0.81 | 0.29 |  |  | 0.25 | 1.37 |
| =4 | 1.97 | 0.53 |  |  | 0.94 | 3.00 |
| **Item 1 – Auditory** | | | | | | |
| Discrim. difference | 2.46 | 0.80 | 3.08 | 0.002 | 0.90 | 4.03 |
| >=2 | -0.58 | 0.27 |  |  | -1.11 | -0.05 |
| =4 | 0.77 | 0.29 |  |  | 0.20 | 1.34 |
| **Item 8 – Tactile – Nasal swab-** | | | | | | |
| Discrim. difference | 2.27 | 0.77 | 2.94 | 0.003 | 0.76 | 3.78 |
| >=2 | -0.06 | 0.24 |  |  | -0.52 | 0.41 |
| =4 | 1.55 | 0.45 |  |  | 0.67 | 2.42 |
| **Item 5 - Threat** | | | | | | |
| Discrim. difference | 2.24 | 0.84 | 2.65 | 0.008 | 0.58 | 3.89 |
| >=2 | -0.06 | 0.24 |  |  | -0.53 | 0.41 |
| =4 | 0.29 | 0.25 |  |  | -0.20 | 0.78 |
| **Item 7 – Tactile – Shoulder tap-** | | | | | | |
| Discrim. difference | 1.85 | 0.67 | 2.78 | 0.005 | 0.54 | 3.16 |
| >=2 | -1.20 | 0.42 |  |  | -2.02 | -0.38 |
| =4 | -0.44 | 0.28 |  |  | -0.99 | 0.10 |
| **Item 6 - Olfactory** | | | | | | |
| Discrim. difference | 1.40 | 0.51 | 2.73 | 0.006 | 0.39 | 2.40 |
| >=2 | -1.84 | 0.62 |  |  | -3.05 | -0.62 |
| =4 | -0.08 | 0.30 |  |  | -0.67 | 0.51 |
| **Item 10 – Pain – Ear pinch-** | | | | | | |
| Discrim. difference | 1.18 | 0.48 | 2.43 | 0.015 | 0.23 | 2.12 |
| >=2 | 0.35 | 0.34 |  |  | -0.32 | 1.03 |
| =4 | 2.30 | 0.86 |  |  | 0.61 | 3.99 |
| **Item 11 - Vocalization** | | | | | | |
| Discrim. difference | 0.68 | 0.47 | 1.45 | 0.148 | -0.24 | 1.59 |
| >=2 | -4.60 | 3.04 |  |  | -10.56 | 1.35 |
| =4 | -1.48 | 1.07 |  |  | -3.59 | 0.62 |

**IRT Model – Graded response model for all CNCS assessments**

Results are sorted by descending discrimination power.

Graded response model

Number of observations = 212

Iterations to convergence = 5

Log likelihood = -1753.04

|  | Coefficient | Std. err. | Z | P>\|z\| | [95% confidence interval] | |
| --- | --- | --- | --- | --- | --- | --- |
| **Item 3 - Visual – Fixation or avoidance** | | | | | | |
| Discrim. difference | 3.52 | 0.59 | 5.94 | <0.001 | 2.36 | 4.69 |
| >=2 | -0.66 | 0.11 |  |  | -0.87 | -0.45 |
| =4 | 0.00 | 0.09 |  |  | -0.18 | 0.18 |
| **Item 4 - Visual – Fixation & tracking** | | | | | | |
| Discrim. difference | 3.48 | 0.57 | 6.11 | <0.001 | 2.36 | 4.60 |
| >=2 | -0.50 | 0.10 |  |  | -0.70 | -0.30 |
| =4 | 0.20 | 0.09 |  |  | 0.01 | 0.38 |
| **Item 5 - Threat** | | | | | | |
| Discrim. difference | 2.94 | 0.47 | 6.28 | <0.001 | 2.02 | 3.85 |
| >=2 | 0.02 | 0.10 |  |  | -0.17 | 0.21 |
| =4 | 0.43 | 0.10 |  |  | 0.23 | 0.63 |
| **Item 7 - Tactile – Shoulder tap-** | | | | | | |
| Discrim. difference | 2.58 | 0.40 | 6.36 | <0.001 | 1.78 | 3.37 |
| >=2 | -0.87 | 0.13 |  |  | -1.12 | -0.62 |
| =4 | -0.32 | 0.10 |  |  | -0.53 | -0.12 |
| **Item 1 - Auditory** | | | | | | |
| Discrim. difference | 2.39 | 0.34 | 7.09 | <0.001 | 1.73 | 3.05 |
| >=2 | -0.37 | 0.11 |  |  | -0.58 | -0.15 |
| =4 | 1.00 | 0.13 |  |  | 0.73 | 1.26 |
| **Item 2 - Command responsivity** | | | | | | |
| Discrim. difference | 2.36 | 0.35 | 6.77 | <0.001 | 1.68 | 3.05 |
| >=2 | -0.64 | 0.12 |  |  | -0.87 | -0.40 |
| =4 | 0.03 | 0.10 |  |  | -0.17 | 0.24 |
| **Item 9 - Pain – Firm pinch finger tip-** | | | | | | |
| Discrim. difference | 2.03 | 0.48 | 4.18 | <0.001 | 1.08 | 2.98 |
| >=2 | 1.73 | 0.25 |  |  | 1.24 | 2.21 |
| =4 | 2.80 | 0.46 |  |  | 1.90 | 3.70 |
| **Item 8 - Tactile – Nasal swab-** | | | | | | |
| Discrim. difference | 1.27 | 0.21 | 5.91 | <0.001 | 0.85 | 1.69 |
| >=2 | 0.28 | 0.14 |  |  | 0.00 | 0.57 |
| =4 | 2.14 | 0.33 |  |  | 1.50 | 2.78 |
| **Item 10 - – Pain – Ear pinch-** | | | | | | |
| Discrim. difference | 1.07 | 0.21 | 5.12 | <0.001 | 0.66 | 1.48 |
| >=2 | 0.94 | 0.20 |  |  | 0.54 | 1.34 |
| =4 | 2.66 | 0.47 |  |  | 1.75 | 3.57 |
| **Item 6 - Olfactory** | | | | | | |
| Discrim. difference | 0.96 | 0.18 | 5.31 | <0.001 | 0.60 | 1.31 |
| >=2 | -2.42 | 0.44 |  |  | -3.28 | -1.56 |
| =4 | 0.10 | 0.17 |  |  | -0.24 | 0.44 |
| **Item 11 - Vocalization** | | | | | | |
| Discrim. difference | 0.77 | 0.18 | 4.20 | <0.001 | 0.41 | 1.12 |
| >=2 | -3.54 | 0.81 |  |  | -5.13 | -1.94 |
| =4 | 0.59 | 0.24 |  |  | -1.06 | -0.12 |

**Boundary characteristic curves for CNCS items**

First assessment	All assessments

**Category characteristic curves for CNCS items**

First assessment	All assessments

**Test characteristic curves for CNCS (all items)**

First assessment	All assessments

**Test Information functions for CNCS (all items)**

First assessment	All assessments

**Item Information functions for CNCS subscales**

First assessment	All assessments

**IRT Model – Graded response model for first CNCS assessment, based uniquely on the CNCS level (i.e., not on the single items).**

Results are sorted by descending discrimination power.

Graded response model

Number of observations = 40

Iterations to convergence = 3

Log likelihood = -59.62

|  | Coefficient | Std. err. | Z | P>\|z\| | [95% confidence interval] | |
| --- | --- | --- | --- | --- | --- | --- |
| **Level** | | | | | | |
| Discrim. difference | 0.73 | 19.43 | 0.04 | 0.970 | -37.36 | 38.81 |
| >=1 | -2.62 | 57.64 |  |  | -115.59 | 110.36 |
| >=2 | -0.95 | 20.39 |  |  | -40.91 | 39.01 |
| >=3 | 1.48 | 31.99 |  |  | -61.22 | 64.17 |
| =4 | 3.74 | 84.30 |  |  | -161.49 | 168.97 |

**IRT Model – Graded response model for all CNCS assessments, based uniquely on the CNCS level (i.e., not on the single items).**

Results are sorted by descending discrimination power.

Graded response model

Number of observations = 212

Iterations to convergence = 4

Log likelihood = -306.73

|  | Coefficient | Std. err. | Z | P>\|z\| | [95% confidence interval] | |
| --- | --- | --- | --- | --- | --- | --- |
| **Level** | | | | | | |
| Discrim. difference | 0.79 | 19.69 | 0.04 | 0.968 | -37.80 | 39.37 |
| >=1 | -2.17 | 43.34 |  |  | -87.11 | 82.78 |
| >=2 | -0.11 | 2.14 |  |  | -4.31 | 4.09 |
| >=3 | 1.83 | 36.47 |  |  | -69.64 | 73.31 |
| =4 | 4.86 | 103.58 |  |  | -198.16 | 207.87 |

**Boundary characteristic curves for CNCS level**

First assessment	All assessments

**Category characteristic curves for CNCS level**

First assessment	All assessments

**Test characteristic curves for CNCS level**

First assessment	All assessments

**Test Information functions for CNCS level**

First assessment	All assessments

**Comparisons: Traumatic vs. Non-traumatic cases**

**Test characteristic curves**

All CRS-R assessments	All CNCS assessments*

* the curves for the two groups (‘traumatic’ and ‘non traumatic’) are identical. Hence, only one is visible in the graph.
